# Pediatric Rhabdomyosarcoma in the Head and Neck: An Overview

**DOI:** 10.1055/a-2907-5254

**Published:** 2026-08-17

**Authors:** Michael Sabbaj, Katrina Winsnes, Mathew Geltzeiler

**Affiliations:** 1 12352 Larner College of Medicine, The University of VermontBurlingtonVermontUnited States; 2 Division of Hematology and Oncology 6684 Oregon Health & Science UniversityPortlandOregonUnited States; 3 Department of Otolaryngology-Head and Neck Surgery 6684 Oregon Health & Science UniversityPortlandOregonUnited States

**Keywords:** pediatric rhabdomyosarcoma, sinonasal tumor, parameningeal rhabdomyosarcoma, embryonal rhabdomyosarcoma, salvage therapy, radiation therapy, skull base tumor, pediatric head and neck cancer, recurrent rhabdomyosarcoma, delayed primary excision

## Abstract

Sinonasal pediatric rhabdomyosarcoma is a rare cancer with a prognosis dependent on stage/grade, histology, and molecular characteristics. Radiation therapy is considered first-line therapy in conjunction with chemotherapy, determined based on the disease risk group. Surgery is generally recommended when margin-free resections are possible. Here we present a unique case report of a patient with recurrent rhabdomyosarcoma and an overview of the salient literature pertaining to the management of this case.

## Introduction

Pediatric rhabdomyosarcoma (RMS) comprises 7% of all pediatric cancers with approximately 40% of cases arising in the head and neck (H&N). 1 2 Prognosis and treatment options vary greatly depending on the location, stage/grade, histology, and genetics.

## Case Report

**Fig. 1:**
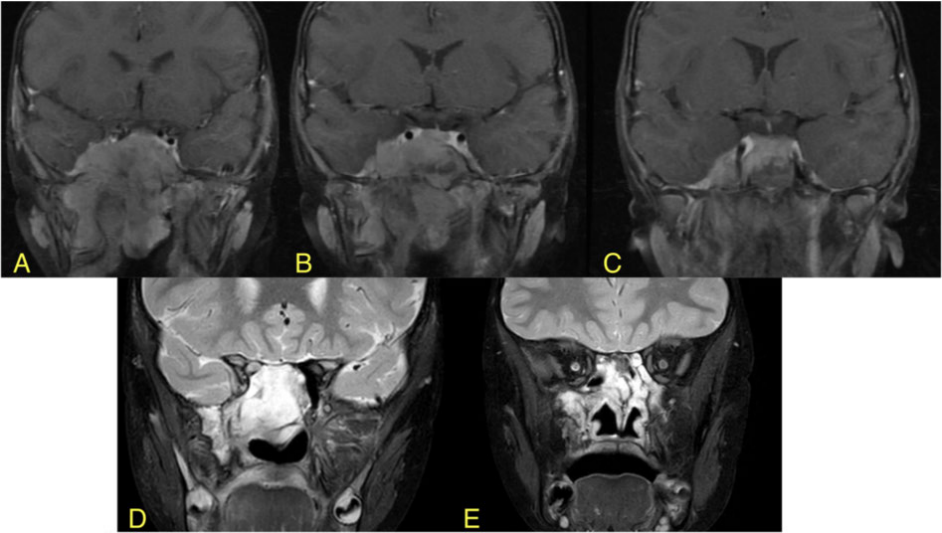
Serial coronal MRI images demonstrating treatment response over time. ( **A–C** ) Pre-treatment MRI demonstrating an enhancing sinonasal mass with extension into adjacent skull base structures. ( **D** ) Post-chemotherapy MRI showing interval decrease in tumor burden. ( **E** ) Post-radiation MRI demonstrating further treatment response of the previously visualized mass.

**Fig. 2:**
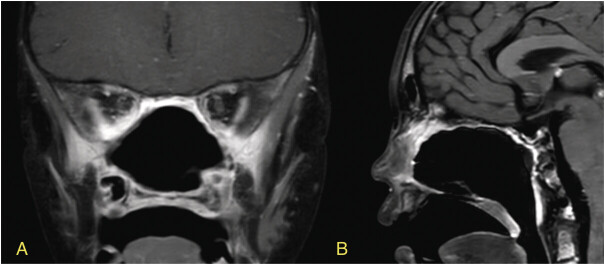
Postoperative MRI demonstrating the surgical cavity following definitive resection of a sinonasal tumor. Coronal ( **A** ) and sagittal ( **B** ) views showing expected postoperative and reconstructive changes with no evidence of residual enhancing mass.

An 8-year-old male presented with locally recurrent, stage 3, parameningeal embryonal rhabdomyosarcoma (ERMS) 15 months after completion of initial therapy. MRI demonstrated a mass involving the central skull base and sphenoid bone with extension into the right pterygopalatine fossa (PPF), cranial fossa, posterior orbit, and ethmoid sinuses ( Fig. 1 ). He underwent salvage chemotherapy and re-irradiation with photons followed by endoscopic resection of recurrent skull base parameningeal RMS after prior surgery and neoadjuvant radiotherapy ( Fig. 2 ). A left medial maxillectomy enabled a transpterygoid approach to the pterygopalatine fossa, with internal maxillary artery ligation, vidian neurectomy, and mobilization of tumor from the nasal cavity, maxillary sinus, nasopharynx, and skull base. Resection included involved nasal cavity and nasopharyngeal mucosa with partial posterior septal resection to optimize margins. The infraorbital nerve was traced to foramen rotundum, and extradural dissection was performed along Meckel’s cave and middle cranial fossa. Frozen margins were negative, and no CSF leak occurred. His disease has been stable with continued salvage chemotherapy and multiple surgical resections, though he remains high risk.

## Literature Review

Several different subtypes of rhabdomyosarcoma (RMS) have been categorized, including embryonal, alveolar (ARMS), spindle cell, and pleomorphic. 3 ERMS is the most common and typically affects children under 5. ARMS is less differentiated and more aggressive than the other subtypes and is more common in adolescents. 4 Spindle cell RMS is very rare, while pleomorphic RMS is typically treated as a soft tissue sarcoma. 5

RMS classification and staging utilize a unique grading system to predict prognosis. The Intergroup Rhabdomyosarcoma Study Group (IRSG) outlines four clinical subgroups based on the extent of disease and initial resection. 6 7 8 H&N RMS additionally incorporates location into the grade: parameningeal (PM), non-parameningeal (non-PM), and orbital. Within PM, the paranasal sinuses, infratemporal fossa, and PPF are particularly unfavorable locations. 5 9 10

Outcomes for patients with metastatic disease or relapses are poor. 11 12 13 The standard chemotherapy regimen for RMS in North America includes vincristine, dactinomycin, cyclophosphamide, and sometimes irinotecan (VAC). 13 14 Doxorubicin was associated with higher adverse effects and did not significantly improve overall survival. 15

Wen et al demonstrated that patients with H&N RMS who received radiation therapy (RT) as part of the initial therapy had improved overall survival (OS) and event free survival compared with those without (80.3% vs. 49.7%). Those who received salvage RT had a better 5-year OS compared with those who did not (66.0% vs. 31.2%). 16 Koscielniak et al reported proton and photon RT had comparable results. 17

Patients with PM ERMS who achieve complete surgical resection may be able to avoid RT. Surgery is only recommended if a margin-free resection is possible. However, there is preliminary data to suggest delayed primary excision (DPE) after induction chemotherapy and radiation for local control may be beneficial, but this remains a field of active investigation. 18 19 The decision to pursue DPE must weigh the potential morbidity of surgery versus the increased radiation dose.
